# The impact of COVID‐19 on heart failure hospitalization and management: report from a Heart Failure Unit in London during the peak of the pandemic

**DOI:** 10.1002/ejhf.1925

**Published:** 2020-07-04

**Authors:** Daniel I. Bromage, Antonio Cannatà, Irfan A. Rind, Caterina Gregorio, Susan Piper, Ajay M. Shah, Theresa A. McDonagh

**Affiliations:** ^1^ School of Cardiovascular Medicine and Sciences King's College London British Heart Foundation Centre of Excellence, James Black Centre London UK; ^2^ Department of Cardiology King's College Hospital London London UK; ^3^ Biostatistics Unit University of Trieste Trieste Italy; ^4^ NICOR National Heart Failure Audit Clinical Lead London UK

**Keywords:** Acute heart failure, COVID‐19, Coronavirus, Hospitalization, Management, National Heart Failure Audit

## Abstract

**Aims:**

To examine the impact of COVID‐19 on acute heart failure (AHF) hospitalization rates, clinical characteristics and management of patients admitted to a tertiary Heart Failure Unit in London during the peak of the pandemic.

**Methods and results:**

Data from King's College Hospital, London, reported to the National Heart Failure Audit for England and Wales, between 2 March–19 April 2020 were compared both to a pre‐COVID cohort and the corresponding time periods in 2017 to 2019 with respect to absolute hospitalization rates. Furthermore, we performed detailed comparison of patients hospitalized during the COVID‐19 pandemic and patients presenting in the same period in 2019 with respect to clinical characteristics and management during the index admission. A significantly lower admission rate for AHF was observed during the study period compared to all other included time periods. Patients admitted during the COVID‐19 pandemic had higher rates of New York Heart Association III or IV symptoms (96% vs. 77%, *P* = 0.03) and severe peripheral oedema (39% vs. 14%, *P* = 0.01). We did not observe any differences in inpatient management, including place of care and pharmacological management of heart failure with reduced ejection fraction.

**Conclusion:**

Incident AHF hospitalization significantly declined in our centre during the COVID‐19 pandemic, but hospitalized patients had more severe symptoms at admission. Further studies are needed to investigate whether the incidence of AHF declined or patients did not present to hospital while the national lockdown and social distancing restrictions were in place. From a public health perspective, it is imperative to ascertain whether this will be associated with worse long‐term outcomes.

## Introduction

Acute heart failure (AHF) is a life‐threatening condition that typically mandates admission to hospital, urgent investigation and treatment. In England and Wales, AHF was cited as the primary diagnosis for more than 90 000 admissions in 2017 to 2018 and was associated with in‐hospital mortality of 10.1%.^1^ Furthermore, among patients surviving to discharge, 1‐year mortality was 32%.^1^ There are several, well‐established factors associated with 1‐year post‐discharge mortality, broadly grouped into three categories: patient characteristics reflecting the severity of heart failure (HF) [e.g. age, New York Heart Association (NYHA) class, left ventricular function], hospitalization factors [including specialist cardiology input, initiation of therapy for HF with reduced ejection fraction (HFrEF) and length of stay] and follow‐up arrangements.^1^ It has been shown repeatedly that 1‐year mortality is lower for patients admitted to cardiology wards compared to general medical wards, as well as for those receiving specialist multi‐disciplinary HF management and receiving optimal medical therapy for HFrEF during the admission.^1^, ^2^

The COVID‐19 pandemic is a global public health emergency. The high burden on healthcare systems, including the National Health Service (NHS) in England and Wales, has mandated rapid redirection of services to manage the influx of patients with COVID‐19. This is especially true in London, which has experienced the highest burden of COVID‐19 in the UK. This transformation has included cessation of routine treatment and face‐to‐face follow‐up of many conditions to both reduce the risk of infection to at‐risk groups and create extra capacity of both personnel and beds. Furthermore, there has been a concurrent decline in patients presenting with many acute medical conditions, including acute coronary syndromes (ACS).^3^, ^4^ To date, little is known about whether similar trends are apparent in AHF, or whether the patient, hospitalization and follow‐up factors that predict outcomes have changed during the current crisis. It is also not known if COVID‐19 has contributed to an increase in AHF admissions, as respiratory illness is well known to contribute to HF decompensation.^5^

Therefore, the present study compared patients presenting with AHF to a tertiary Heart Failure Unit in a London teaching hospital in March–April 2020 to the same period in 2019. We aimed to compare these cohorts with respect to factors associated with outcome, described above, including absolute hospitalization rates, patient characteristics and management during the index admission. We hypothesized that the incidence of hospitalization for AHF would decline during the COVID‐19 pandemic and that inpatient management and follow‐up arrangements would be worse during the pandemic compared to the corresponding period in 2019.

## Methods

### Study design and population

The numbers of consecutive patients hospitalized for AHF in King's College Hospital between 2 March 2020, the date of the first coronavirus death in England, and 19 April were compared to hospitalization numbers over the same number of weeks before the study period and corresponding time periods in 2017 to 2019. Next, we performed detailed comparison of patients hospitalized during the COVID‐19 pandemic and patients presenting in the same period in 2019. Finally, we examined hospital admissions with a diagnosis of HF in the second position, comparing the study period to the same period in 2019, to ascertain whether patients were admitted with AHF secondary to COVID‐19.

The Heart Failure Unit at this hospital provides comprehensive multi‐disciplinary HF care in the inpatient and outpatient settings, including: an electronic referral service to allow daily review of all admissions to hospital with HF by a consultant cardiologist (irrespective of their place of admission), nurse education for inpatients, advice regarding optimal medical therapy and facilitation of follow‐up post discharge. This service operated normally during the pandemic period. All patients are admitted to the hospital via the emergency department. Patients <18 years of age were excluded from the analysis.

Variables were extracted from local National Heart Failure Audit (NHFA) data. The NHFA collects data relating to AHF hospitalizations from NHS Trusts in England and Health Boards in Wales. Patients were entered into the audit if they had a primary discharge diagnosis of AHF, based on appropriate ICD codes [I11.0 Hypertensive heart disease with (congestive) heart failure; I25.5 Ischaemic cardiomyopathy; I42.0 Dilated cardiomyopathy; I42.9 Cardiomyopathy, unspecified; I50.0 Congestive heart failure; I50.1 Left ventricular failure; I50.9 Heart failure, unspecified], and these data were interrogated for the study.

### Data fields

Mandatory fields in the NHFA were collected, including demographics, presenting symptoms and signs, comorbidities, diagnostic tests, place of care, specialist input, length of stay, prescribing for HFrEF and in‐hospital mortality. The standard dataset used for the NHFA is available from NICOR (https://www.nicor.org.uk/national-cardiac-audit-programme/datasets).

### Primary and secondary outcomes

The primary outcome was the rate of hospitalization for AHF. Secondary outcomes were place of care, rates of specialist input, discharge medication for HFrEF and in‐hospital mortality.

### Statistical analyses

Demographic and baseline characteristics of the study populations were compared using the Pearson Chi‐square test for categorical variables, Student's *t*‐test (parametric) or Mann–Whitney U test (non‐parametric) for continuous variables, or one‐way ANOVA for multiple comparisons. Normality of distribution was assessed by the Shapiro–Wilks test. Continuous variables were reported as means (standard deviation) or median [interquartile range (IQR)], as appropriate. Categorical variables were reported as numbers (percentage). Incidence rates for the primary outcome (HF hospitalizations per 100 000 people per week) were calculated by dividing the number of cumulative events per week by the catchment population of King's College Hospital. Incidence rate ratios comparing the exposed (2020) to the unexposed (2019) period were calculated using Poisson regression to model the number of HF hospitalizations per week. A quadratic term was used to fit hospitalization trends in both the exposed and unexposed cohorts and compared by means of the extra‐sum‐of‐square F test. Gaussian fitting was employed to plot the daily number of COVID‐19 cases. All analyses were conducted using SPSS statistics software, version 20.0 (IBM Corp., Armonk, NY, USA), Stata software, version 13.1 (StataCorp., College Station, TX, USA) and ‘R’ (R Project for Statistical Computing).

## Results

**Figure 1:**
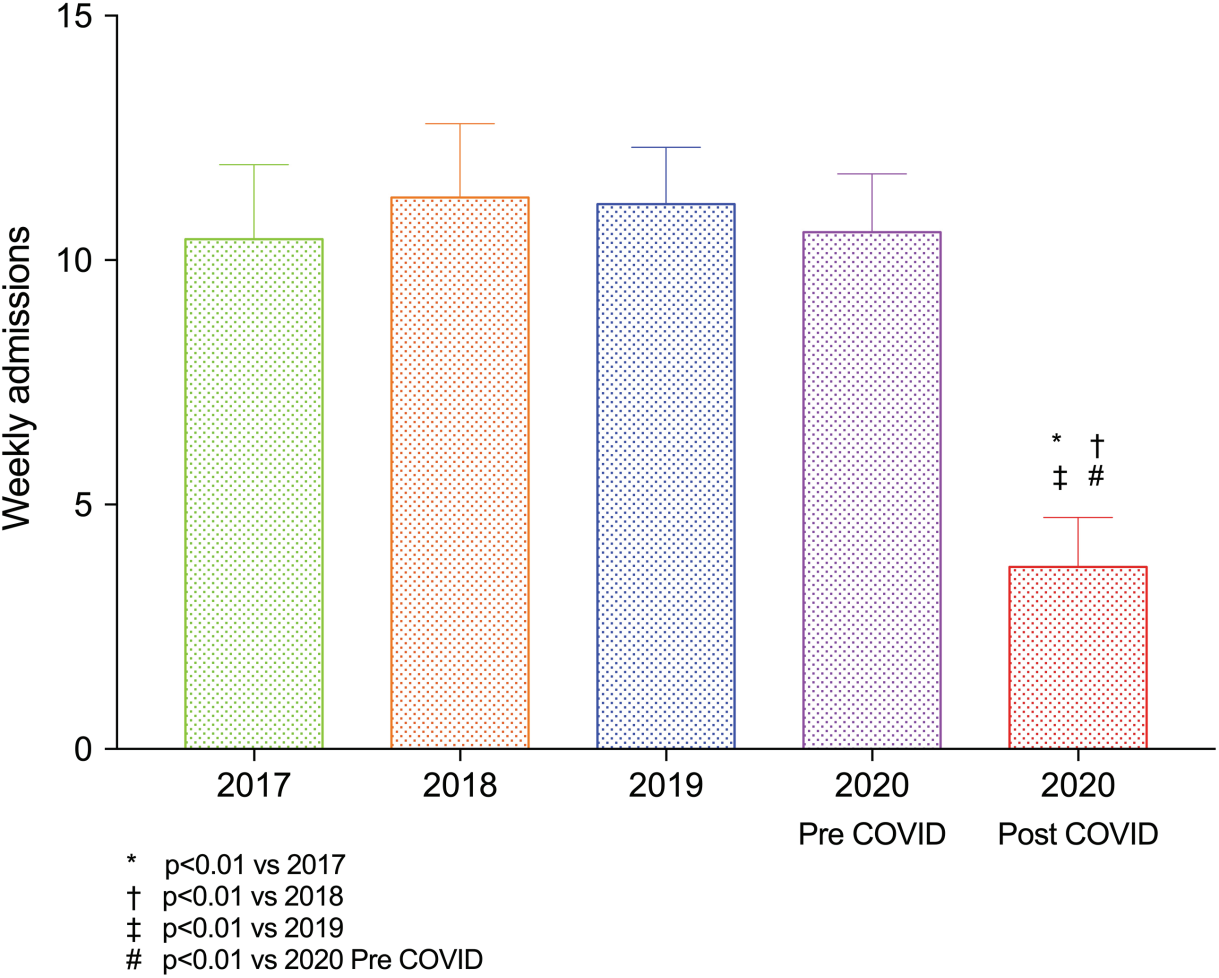
Weekly admissions for heart failure pre‐ and post‐COVID‐19 and in corresponding time periods in 2017 to 2019.

**Figure 2:**
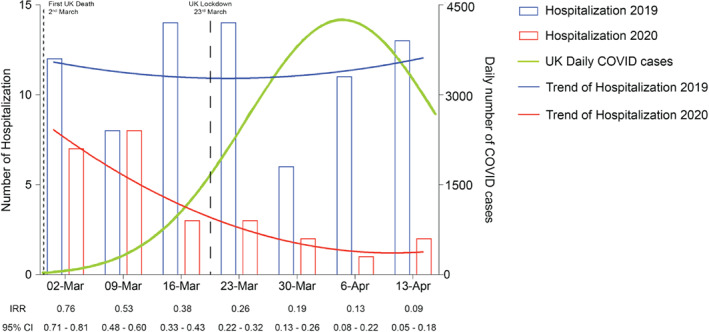
Numbers and trends of heart failure hospitalization by year. CI, confidence interval; IRR, incidence rate ratio.

During the study period, we identified 26 patients hospitalized in King's College Hospital with AHF as the primary diagnosis, which was significantly lower than the number of admissions in the pre‐COVID period, or any of the years 2017 to 2019 (*Figure* *1*). Furthermore, the relative risk of hospitalization decreased over time compared to the corresponding time period in 2019 (*Figure* *2*). No patients with a primary diagnosis of AHF also had a diagnosis of COVID‐19.

Patient baseline characteristics according to exposure group are shown in *Table* *1*. Patients had similar mean age at AHF admission (73 ± 14 years in 2020 vs. 71 ± 15 years in 2019), similar proportions of women (46% vs. 42%) and similar ethnicity (42% vs. 39%). Overall, comorbidities and cardiovascular risk factors were similar between patients presenting in each year (*Table* *1*), although fewer patients in 2020 had pre‐existing, significant valve disease (27% vs. 51%, *P* = 0.04). Furthermore, similar proportions presented with decompensated HFrEF (69% vs. 63%) and HF with preserved ejection fraction (31% vs. 37%) and this is consistent with overall NHFA data.^1^

**Table 1:** Baseline characteristics by year of presentation

	2019 (*n* = 78, 75%)	2020 (*n* = 26, 25%)	*P*‐value
Age (years), mean (SD)	71 (15)	73 (14)	NS
Male sex, *n* (%)	45 (58)	14 (54)	NS
Height (cm), mean (SD)	167 (9)	167 (9)	NS
Race, *n* (%)			NS
White	41 (53)	14 (54)	
Black	30 (39)	11 (42)	
Other	7 (9)	1 (4)	
Admission BMI (kg/m^2^), mean (SD)	30 (9)	31 (8)	NS
Admission heart rate (bpm), mean (SD)	88 (24)	86 (25)	NS
Admission rhythm, *n* (%)			
Sinus rhythm	41 (54)	10 (42)	NS
Atrial fibrillation	29 (38)	12 (50)	NS
Admission blood pressure (mmHg), mean (SD)			
Systolic	134 (26)	140 (30)	NS
Diastolic	79 (17)	78 (21)	NS
NYHA class, *n* (%)			0.01
I	4 (5)	0 (0)	
II	14 (18)	1 (4)	
III	41 (53)	10 (38)	
IV	19 (24)	15 (58)	
Severity of oedema, *n* (%)			0.02
None	10 (13)	5 (19)	
Mild	19 (24)	5 (19)	
Moderate	38 (49)	6 (23)	
Severe	11 (14)	10 (39)	
HF classification at admission, *n* (%)			
HFrEF	48 (63)	18 (69)	NS
HFpEF	28 (37)	8 (31)	
Comorbidities, *n* (%)			
Ischaemic heart disease	29 (37)	8 (31)	NS
Acute myocardial infarction	8 (10)	5 (19)	NS
Valve disease	40 (51)	7 (27)	0.04
Hypertension	54 (69)	21 (81)	NS
Diabetes	39 (50)	14 (54)	NS
Asthma	7 (9)	2 (8)	NS
COPD	16 (21)	5 (19)	NS
Device	13 (17)	9 (35)	NS

BMI, body mass index; COPD, chronic obstructive pulmonary disease; HF, heart failure; HFpEF, heart failure with preserved ejection fraction; HFrEF, heart failure with reduced ejection fraction; NYHA, New York Heart Association; SD, standard deviation.

We observed that patients hospitalized in 2020 had a higher proportion with NYHA class III or IV symptoms (96% vs. 77%, *P* = 0.03) and severe peripheral oedema (39% vs. 14%, *P* = 0.01; *Table* *1*).

In‐hospital management of patients was similar in our centre in 2019 and 2020. Fewer patients were admitted to cardiology wards (23% vs. 37%) or reviewed by HF specialists (56% vs. 78%) in 2020 compared to 2019, but these findings were not significant (*Table* *2*). Moreover, length of stay [6 (IQR 4–9) days in 2020 vs. 7 (IQR 3–13) days in 2019] and multi‐disciplinary HF team follow‐up (31% in 2020 vs. 28% in 2019) were unchanged between the two cohorts. We did not observe any differences in discharge medication between 2019 and 2020, with similar proportions being discharged on evidence‐based therapy for HFrEF (*Table* *3*). Furthermore, we observed no difference in discharge daily diuretic dose (119 ± 83 mg in 2020 vs. 108 ± 77 mg in 2019, *P* = 0.55). In‐hospital mortality was very low in both years, which precluded statistical comparison. Our inpatient mortality rates have been significantly lower than the NHFA average for the last 8 years.

**Table 2:** In‐hospital management by year of presentation

	2019 (*n* = 78, 75%)	2020 (*n* = 26, 25%)	*P*‐value
Place of care, *n* (%)			NS
Cardiology	29 (37)	6 (23)	
General medicine	40 (51)	19 (73)	
Other	9 (12)	1 (4)	
Specialist input, *n* (%)	61 (78)	15 (56)	NS
Discharge bloods, mean (SD)			
Haemoglobin (g/L)	119 (22)	115 (17)	NS
Urea (mmol/L)	11 (8)	12 (7)	NS
Creatinine (μmol/L)	130 (83)	144 (66)	NS
Sodium (mmol/L)	138 (4)	139 (3)	NS
Potassium (mmol/L)	4.2 (0.6)	4.3 (0.5)	NS
Weight change (kg), mean (SD)	−3 (3)	−3 (4)	NS
Length of stay (days), median [IQR]	7 [3–13]	6 [4–9]	NS
Multidisciplinary HF team follow‐up	22 (28)	8 (31)	NS
Died in hospital, *n* (%)	2 (2)	1 (4)	NS

HF, heart failure; IQR, interquartile range; SD, standard deviation.

**Table 3:** Discharge medication for patients with heart failure with reduced ejection fraction by year of presentation

Discharge medication	2019 (*n* = 48, 73%)	2020 (*n* = 18, 27%)	*P*‐value
ACEI or ARB	37 (77)	15 (83)	NS
Beta‐blocker	43 (90)	14 (78)	NS
Diuretic	45 (94)	17 (94)	NS
MRAs	27 (56)	10 (56)	NS
ACEI or ARB, beta‐blocker and MRA	20 (42)	7 (39)	NS
Digoxin	10 (21)	3 (18)	NS

Values are given as *n* (%).

ACEI, angiotensin‐converting enzyme inhibitor; ARB, angiotensin receptor blocker; MRA, mineralocorticoid receptor antagonist.

**Figure 3:**
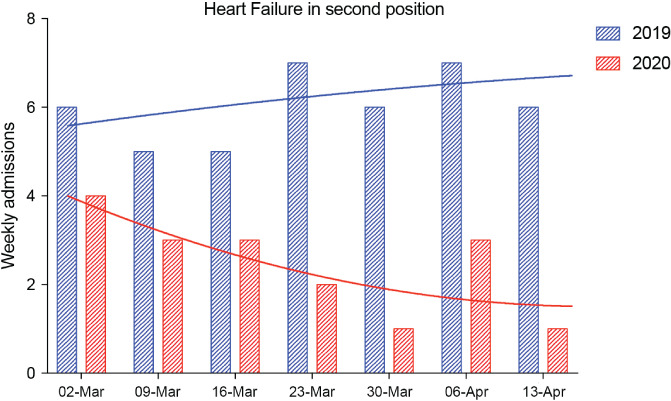
Numbers and trends of heart failure diagnoses in the second diagnostic position by year.

Finally, one patient had COVID‐19 as a primary diagnosis and HF in the second diagnostic position. We identified seven patients hospitalized with HF as the secondary diagnosis during the study period, which was significantly lower than the 52 seen in 2019 (*P* = 0.004; *Figure* *3*).

## Discussion

While the impact of the COVID‐19 pandemic on ACS has been reported,^3^, ^4^ little is known about its effects on the admission and management of patients with AHF. We report a significant decline in hospitalization rates for AHF during the COVID‐19 pandemic, compared to before the pandemic and each of the preceding 3 years. Compared to the same period in 2019, the relative risk of hospitalization for AHF progressively reduced during the COVID‐19 pandemic. The same was true for HF diagnoses in the second position and only one patient had a co‐existing diagnosis of COVID‐19, despite the known increase of COVID‐19 among patients with cardiovascular comorbidity.^6^ It is unknown if the incidence of AHF has declined or if our findings reflect a reluctance to attend hospital, although similar reports on ACS suggest the latter.^4^

We observed a higher proportion of black patients than are reported in most AHF cohorts, which reflects our local population (for example, a study using Clinical Practice Research Datalink reported 96.1% of incident HF cases to be among Caucasians^7^). Compared to 2019, patients were, in general, more unwell with higher rates of NYHA class III or IV symptoms and severe peripheral oedema, which are known predictors of poor outcomes in AHF.^8^, ^9^ Following admission, in‐hospital management was similar between the two cohorts. We did not observe a difference in discharge daily diuretic dose, although this may not reflect in‐hospital treatment. Similarly, we observed no significant difference in rates of specialty input, main place of care, length of stay or pharmacological management of HFrEF. This reflects the fact that we continued to provide our comprehensive inpatient care model during the pandemic. However, despite this, our early experience suggests a trend towards more patients being managed on general medical wards and fewer receiving specialist HF input.

Taken together, our data might suggest that patients with less severe AHF have avoided presenting to hospital, although we were unable to confirm this in our study. The national lockdown and social distancing restrictions may have reduced respiratory tract infections, which are a common trigger for HF decompensation. However, it is unlikely that this would account for a decline only in patients with milder symptoms. While there is no evidence for the outcome of patients with decompensated HF managed in the community, it is expected that this will translate to a subsequent increase in HF hospitalization and mortality. This is likely to be exacerbated by the known decline in ACS presentations during this period that will no doubt lead to an increase in the prevalence and incidence of HF in the months to come.

Based on these findings, it is imperative to carefully examine longitudinal primary care, hospitalization and mortality data for HF to ascertain the reasons for, and implications of, the shift towards fewer, sicker patients being hospitalized for AHF.^10^ It is likely that such analyses, including comparison of different models of care during the COVID‐19 pandemic, can help inform our response to future public health emergencies.

### Strengths and limitations

The NHFA is a well established registry that sets the performance standards for hospitals as to what constitutes optimal HF care, as well as being the mechanism for remuneration of hospitals achieving the key performance indicators with the Best Practice Tariff (for submission of at least 70% of primary admissions with HF to the audit and making sure that 60% of those receive specialist care during the admission). Furthermore, as a single‐centre study, our dataset is accurate and reliable with low levels of missing data. However, this work has the usual limitations of observational data, including potential bias as a result of unmeasured residual confounding, and the fact that observed differences may be due to unmeasured variables. Moreover, the results may not be generalizable to other Heart Failure Units. We can only report associations with year of presentation, rather than causal relationships, and patients presenting at different times may differ with respect to unrecorded variables.

## Conclusion

We report a decline in AHF hospitalization in our centre during the COVID‐19 pandemic, but hospitalized patients had more severe symptoms. Further studies are needed to investigate whether the incidence of AHF truly declined or patients did not present to hospital while the lockdown and social distancing restrictions were in place. From a public health perspective, it is imperative to ascertain whether this is associated with worse outcomes.

## References

[1] NICOR . *National Heart Failure Audit – 2019 Summary Report (2017/2018 Data)*. Healthcare Quality Improvement Partnership. https://www.nicor.org.uk/wp‐content/uploads/2019/09/Heart‐Failure‐2019‐Report‐final.pdf (5 June 2020).

[2] Carriere C , Stolfo D , Baglio V , Gerloni R , Merlo M , Barbati G , Cannatà A , Biolo G , Sinagra G . Outcome of the multidimensional prognostic index in ultra‐octogenarian patients hospitalized for cardiovascular diseases. J Cardiovasc Med (Hagerstown) 2018;19:536–545. 30119097 10.2459/JCM.0000000000000699

[3] De Filippo O , D'Ascenzo F , Angelini F , Bocchino PP , Conrotto F , Saglietto A , Secco GG , Campo G , Gallone G , Verardi R , Gaido L , Iannaccone M , Galvani M , Ugo F , Barbero U , Infantino V , Olivotti L , Mennuni M , Gili S , Infusino F , Vercellino M , Zucchetti O , Casella G , Giammaria M , Boccuzzi G , Tolomeo P , Doronzo B , Senatore G , Grosso Marra W , Rognoni A , Trabattoni D , Franchin L , Borin A , Bruno F , Galluzzo A , Gambino A , Nicolino A , Truffa Giachet A , Sardella G , Fedele F , Monticone S , Montefusco A , Omedè P , Pennone M , Patti G , Mancone M , De Ferrari GM . Reduced rate of hospital admissions for ACS during Covid‐19 outbreak in northern Italy. N Engl J Med 2020;383:88–89. 32343497 10.1056/NEJMc2009166 PMC7224608

[4] Metzler B , Siostrzonek P , Binder RK , Bauer A , Reinstadler SJ . Decline of acute coronary syndrome admissions in Austria since the outbreak of COVID‐19: the pandemic response causes cardiac collateral damage. Eur Heart J 2020;41:1852–1853. 32297932 10.1093/eurheartj/ehaa314 PMC7184486

[5] Fonarow GC , Abraham WT , Albert NM , Stough WG , Gheorghiade M , Greenberg BH , O'Connor CM , Pieper K , Sun JL , Yancy CW , Young JB ; OPTIMIZE‐HF Investigators and Hospitals . Factors identified as precipitating hospital admissions for heart failure and clinical outcomes: findings from OPTIMIZE‐HF. Arch Intern Med 2008;168:847–854. 18443260 10.1001/archinte.168.8.847

[6] Guan WJ , Ni ZY , Hu Y , Liang WH , Ou CQ , He JX , Liu L , Shan H , Lei CL , Hui DS , du B , Li LJ , Zeng G , Yuen KY , Chen RC , Tang CL , Wang T , Chen PY , Xiang J , Li SY , Wang JL , Liang ZJ , Peng YX , Wei L , Liu Y , Hu YH , Peng P , Wang JM , Liu JY , Chen Z , Li G , Zheng ZJ , Qiu SQ , Luo J , Ye CJ , Zhu SY , Zhong NS ; China Medical Treatment Expert Group for Covid‐19 . Clinical characteristics of coronavirus disease 2019 in China. N Engl J Med 2020;382:1708–1720. 32109013 10.1056/NEJMoa2002032 PMC7092819

[7] Uijl A , Koudstaal S , Direk K , Denaxas S , Groenwold RH , Banerjee A , Hoes AW , Hemingway H , Asselbergs FW . Risk factors for incident heart failure in age‐ and sex‐specific strata: a population‐based cohort using linked electronic health records. Eur J Heart Fail 2019;21:1197–1206. 30618162 10.1002/ejhf.1350 PMC7074015

[8] Shoaib A , Mamas MA , Ahmad QS , McDonagh TM , Hardman SM , Rashid M , Butler R , Duckett S , Satchithananda D , Nolan J , Dargie HJ , Clark AL , Cleland JG . Characteristics and outcome of acute heart failure patients according to the severity of peripheral oedema. Int J Cardiol 2019;285:40–46. 30905515 10.1016/j.ijcard.2019.03.020

[9] Cleland JG , McDonagh T , Rigby AS , Yassin A , Whittaker T , Dargie HJ ; National Heart Failure Audit Team for England and Wales . The National Heart Failure Audit for England and Wales 2008‐2009. Heart 2011;97:876–886. 21173198 10.1136/hrt.2010.209171

[10] Cannata A , Bromage D , McDonagh T . Cardiology after COVID‐19: quo vademus? Eur Heart J Qual Care Clin Outcomes 2020 May 7. 10.1093/ehjqcco/qcaa042 [Epub ahead of print]. PMC7239219 32379892

